# The Hospital Sunday Fund

**Published:** 1907-12-21

**Authors:** 


### THE METROPOLITAN HOSPITAL SUNDAY FUND.

The annual meeting of the constituents of the Hospital Sunday Fund, of which we give a report below, was so well attended as nearly to fill the Egyptian Hall at the Mansion House. It seems extraordinary that the Hospital Sunday Fund should be made the sport of the varying camps into which the anti-vivisectionists are now divided. Each year of late attempts have been made by some section of the anti-vivisectionists to sew dissension among the congregations, and so to prevent the voluntary hospitals from reaping the full benefits they might otherwise receive from the congregational collections. We can only hope that in some way it may be found possible to supply every clergyman and minister of religion with a complete copy of the evidence which has been given before the Anti-Vivisectionist Committee which completed the taking of evidence on December 19, and that they may each arrange to have a copy of such evidence placed in the vestry of their church or chapel, so that everybody interested may have an opportunity of acquainting themselves with the actual facts. Then we are confident no ingenuity will enable any section of the anti-vivisection body to continue this most regrettable agitation against the Hospital Sunday Fund. It is due to Mr. Lewis, who represented the anti-vivisectionists at the meeting, to say that he made his points well and clearly, and is entitled to credit for the manner in which he discharged a difficult, and, we should imagine, a not altogether unpleasant task. He was hopelessly out of order, being evidently unaccustomed to the procedure of public meeting, and did not even understand the effect of taking a report as read. Mr. Sydney Holland, who spoke on behalf of the Sunday Fund Council, was at his best, and the constituents of the Fund, and all interested in hospitals will, we feel sure, appreciate the services he rendered so admirably on this occasion.

### THE ANNUAL MEETING.

The annual meeting of the constituents of the Metropolitan Hospital Sunday Fund was held on Tuesday, 17th instant, at the Mansion House, the Lord Mayor (Sir John Bell), President and Treasurer, presiding.

The Lord Mayor having put from the chair the motion that the report of the Council for 1907 be taken as read and declared carried, the Rev. Lionel Lewis at once rose from the body of the hall and desired to ask a question. The Lord Mayor ruled that he could not do so then. Mr. Lewis, under the misapprehension that the motion just put was for the adoption of the report, and that opportunity for discussion on it was being refused, persisted that he had a right to be heard. After some altercation, accompanied by cries of approval and dissent from hall and platform, Archdeacon Sinclair pointed out Mr. Lewis's mistake, and that his opportunity would arise on the succeeding resolution, whereupon that gentleman resumed his seat.

Archdeacon Sinclair then rose to move : "That the report of the Council for the year 1907 be, and is hereby, received." It gave him peculiar pleasure, he said, to propose that resolution, since they had this year received the largest sum since the foundation of the Fund, this satisfactory position of affairs being largely due to the generosity of the late George Herring. Unfortunately, the collections from the congregations showed a falling-off of £1,848 as compared with 1906, due, doubtless, to the week-end habit in the West End, and to the fact also that there had been a good deal of financial insecurity in London and the South of England.

The Chief Rabbi (Dr. Adler) seconded the motion. The Sunday Fund fulfilled a beneficent purpose in constituting a bond which united all religious denominations, and in stimulating and sustaining a wondrous interest in the hospitals throughout the Metropolis.

The Rev. Hardy Harwood supported the resolution, observing that experience showed that, so far from injuring local objects, the collections roused a sympathy which had a most beneficial reflex action on the congregations, and recommended themselves if only as a matter of policy.

Mr. Pearce Gould, F.R.C.S., supported the resolution. As one having close association with the Fund, and as on the staff of a hospital which had received great support from it since its foundation, he could say that the hospitals not only welcomed the addition to their resources, but also the sympathy the Fund had drawn out and the suggestions made from time to time as to improvements in working arrangements. Reference had been made to the thirty-six years' harmonious working among all denominations; the hospitals themselves were the outcome of the same brotherly feeling. As regarded one particular subject of controversy, the distribution of the funds was made in strict accordance with the report of Lord Justice Fry's Commission, and the hospitals and medical schools had accepted that finding just as fully and frankly as the Fund had done, and were being influenced by it in all their arrangements.

#### The Anti-Vivisection Question.

The Rev. Lionel Lewis then rose to ask his question. He should like to know, he said, why the Committee of Distribution had withheld a grant from the Battersea General Hospital, otherwise known as the Anti-Vivisection Hospital, in spite of a letter addressed to the Lord Mayor saying that his action had given the gravest dissatisfaction to the inhabitants of Battersea.

The Lord Mayor said he understood the question to be in effect: Why did the Committee not give a grant to some particular public institution? The answer to that was that the Committee so decided, and that the Council had since approved of that decision. Obviously no one member of the Committee was competent to say precisely why twenty or thirty gentlemen so decided.

Mr. Lewis said he desired to move: "That, prior to the adoption of the report, the question of a grant to the Battersea General Hospital be referred back to the Distribution Committee." He was perfectly well aware, he declared of the reason for the refusal of this grant, and was amazed that it was not acknowledged. Battersea, he continued, had a population of 200,000, mainly working people. During 1906 over 20,000 people had been treated at the hospital—there was no other within easy reach, and they had had the low death rate of 6. The distribution Committee withheld a grant from the hospital on the ground that certain remedies, the products of vivisection, were not allowed to be used there, and this refusal was made in face of the fact that the attendances had increased since 1903 from 13,000 to 20,000, thus showing that the people had confidence in it.

Mr. Schofield, who said he had no idea that the question was to be raised and no acquaintance with the gentleman who had raised it, seconded the amendment, on the ground that many supporters of the Fund held the view that vivisection was too much practised at hospitals.

The Lord Mayor then read a copy of the resolution of the Committee of Distribution of July 4: "This Committee is of opinion that the Anti-Vivisection Hospital does not comply with the general conditions which they consider should govern any hospital designed for the relief of the sick poor, and therefore they cannot vote it a grant."

### The Defence.

The Hon. Sydney Holland (Chairman of the London Hospital and a member of the Council of the Fund) said he thought it was people of peculiar views like Mr. Lewis, whose ignorance of his subject was almost phenomenal, and who for the second time was intervening to introduce ill-will at their meetings, who were reducing the Fund to the level of a debating society. He would like to give his reasons for being satisfied that they were right in not giving money to this hospital. Let them not be led away by thinking that Mr. Lewis's concern was for the sick poor of Battersea. His concern was because this was an anti-vivisection hospital, and so he thought it ought to be supported. This was a general hospital, and so the poor who went there thought they were getting the best possible treatment, but they were not. It was not fair, because people were poor and ignorant and miserable, to deny them the best treatment obtainable. Did they suppose all these people at Battersea were worshippers of the brown dog? Most of them knew nothing about it. This was a miserable hospital—miserably built, miserably equipped, and the refusal of a grant was because its work was based on considerations not exclusively for the benefit of its patients. One of these days some crank would be starting a hospital only for those with dark hair or something else equally valid. Could they not trust the men who formed the Council of that Fund—men not alone doctors or connected with hospitals—men whose names, leaving out his own, stood high in the estimation of everyone? He considered it was a fraud to open a hospital, to pretend that poor people were to get the best possible treatment, and then to deny them some of the greatest benefits conferred upon the human race. He asked them to read the evidence given before the Vivisection Commission now sitting. Mr. Coleridge, the great anti-vivisectionist agitator, said in evidence: "I do not oppose vivisection if it be so conducted as to cause no pain." And yet this hospital refused to avail itself of results obtained by this means, and the losers were the sick poor.

The amendment was then put and lost by an overwhelming majority, the Lord Mayor observing: "I think even Mr. Lewis will say that's not carried," after which the original resolution was agreed to *nem. con.*

Canon Fleming moved, and it was resolved, that June 21 be fixed for Hospital Sunday, 1908, and that the cordial co-operation of all ministers of religion within the Metropolitan area be again invited in the usual way.

Mr. Henry Morris proposed that the Council for the year 1907 be re-elected for 1908, with the addition of Rev. Canon Curtis, Right Hon. Lord Leith of Fyvie, the Hon. Harry Lawson, Sir James Reid, Bart., G.C.V.O., K.C.B., etc., Sir Thomas Smith, K.C.V.O., F.R.C.S., Douro Hoare, Esq., and Thomas Wakley, Esq., to fill vacancies.

This was agreed to, and, on the motion of Sir Savile Crossley seconded by Lord Stamford, a vote of thanks to the Lord Mayor closed the proceedings.

